# Inhibition of DDX5-Mediated G-Quadruplex Unwinding in the β-Catenin 5′-UTR by Magnesium Isoglycyrrhizinate Alleviates Chemotherapy-Induced Intestinal Injury

**DOI:** 10.34133/research.1044

**Published:** 2026-03-19

**Authors:** Ying Zhang, Wenqiang Fu, Yaohui Wang, Anyuan Wu, Zhenzhen Zhu, Xunkai Yin, Yan Li, Yongdi Sun, Wenpu Shi, Jianming Cheng, Lihong Hu, Jian Liu, Jian Cui

**Affiliations:** ^1^ School of Pharmacy, Nanjing University of Chinese Medicine, Nanjing 210023, China.; ^2^ Affiliated Hospital of Nanjing University of Chinese Medicine, Jiangsu Province Hospital of Chinese Medicine, Nanjing 210029, China.; ^3^ High Magnetic Field Laboratory, Chinese Academy of Sciences, Hefei 230031, China.; ^4^ Jiangsu Key Laboratory for Functional Substance of Chinese Medicine, Stake Key Laboratory Cultivation Base for TCM Quality and Efficacy, School of Pharmacy, Nanjing University of Chinese Medicine, Nanjing 210023, China.; ^5^ State Key Laboratory of Pharmaceutical Biotechnology, School of Life Science, Nanjing University, Nanjing 210023, China.; ^6^ The Second Affiliated Hospital of Shaanxi University of Chinese Medicine, Xianyang 712000, China.; ^7^ China Joint Graduate School of Traditional Chinese Medicine, Suzhou 215105, China.

## Abstract

Chemotherapy-induced gastrointestinal toxicity, particularly intestinal barrier disruption and diarrhea, is a major dose-limiting adverse effect with unclear mechanisms. Here, we identify magnesium isoglycyrrhizinate (MIG) as a novel therapeutic agent that ameliorates 5-fluorouracil (5-FU)- and irinotecan-induced intestinal injury by enhancing epithelial barrier integrity. Using MIG as a molecular probe, we revealed a pathogenic mechanism underlying chemotherapy-induced barrier damage. Integrated chemoproteomics (limited proteolysis–mass spectrometry and thermal proteome profiling) analyses revealed direct binding of MIG to the RNA helicase DDX5 (DEAD-box helicase 5), whose expression is markedly increased upon 5-FU-induced injury. Mechanistically, DDX5 destabilizes the G-quadruplex (G4) structure in the *CTNNB1* (β-catenin) 5′ untranslated region, suppressing β-catenin production and compromising barrier function. Crucially, MIG acts as a novel DDX5 inhibitor that blocks G4 unwinding, thereby restoring β-catenin expression and barrier integrity in a dose-dependent manner. In vivo, MIG outperforms the canonical DDX5 inhibitor supinoxin in mitigating intestinal damage. Taken together, the results of our study not only establish MIG as a promising therapeutic candidate but also delineate the DDX5/*CTNNB1* axis as a targetable pathway for treating chemotherapy-induced barrier dysfunction.

## Introduction

5-Fluorouracil (5-FU) is used in combination with irinotecan (CPT-11, Camptosar) and leucovorin as a first-line chemotherapy regimen for individuals with metastatic colorectal cancer (CRC) [1,2]. Most chemotherapeutic agents, including 5-FU, induce debilitating gastrointestinal complications in a substantial percentage (40% to 80%) of patients with cancer [3], which often leads to delays or termination of the administration of the chemotherapeutic agent, potentially compromising local tumor control and quality of life [4]. Such inflammatory toxicity manifests as intestinal ulcerations characterized by mucositis, epithelial barrier disruption, crypt cell depletion, and systemic inflammation, with clinical symptoms including severe diarrhea, nausea, vomiting, and abdominal pain during/after treatment [5–7]. Among these symptoms, acute diarrhea is the most prevalent gastrointestinal complication, and strategies to reduce the occurrence of this adverse event are urgently needed.

The pathogenesis of chemotherapy-induced colitis at the cellular and molecular levels remains poorly characterized. While current therapeutic approaches primarily target proinflammatory cytokine neutralization and gut microbiota modulation [8–11], emerging evidence has implicated intestinal epithelial barrier dysfunction as a central pathological mechanism in both chemotherapy-induced mucositis and inflammatory bowel disease (IBD) [12,13]. The intestinal epithelial barrier, which consists of rapidly renewing intestinal epithelial cells (IECs) and their intercellular junctions, is highly vulnerable to damage from chemotherapeutic agents [14]. Intestinal epithelial barrier dysfunction is a major cause of diarrhea following chemotherapy. Tight junction and adherens junction protein complexes are critical for maintaining barrier integrity [15]. β-Catenin is a core structural component of adherens junctions. The depletion of membrane β-catenin disrupts E-cadherin/α-catenin/F-actin anchoring and compromises cell–cell adhesion, exacerbating paracellular permeability, whereas nuclear β-catenin– transcription factor 4 transactivates genes (e.g., *MMP7* and *CD44*) encoding proteins that remodel the epithelial monolayer [16]. Dysregulated β-catenin expression is a hallmark of cancer progression and IBD [17,18]. Our recent work revealed 14-3-3ζ as a key chaperone that stabilizes β-catenin. Targeting this protein with the curcumin derivative AI-34 alleviated dextran sulfate sodium (DSS)-induced ulcerative colitis by promoting the integrity of the intestinal epithelium [19]. Although β-catenin is a promising therapeutic target, there are no effective drugs that specifically target β-catenin barrier function without also carrying the risk of oncogenic activation.

RNA binding proteins (RBPs) are essential for the maintenance of intestinal homeostasis [20]. For example, RNA helicase melanoma differentiation-associated gene 5 gain-of-function variants are associated with early-onset IBD [21]. DDX5 (DEAD-box helicase 5) is among the most abundantly expressed DEAD-box RNA helicases in the intestinal epithelium and exerts a proinflammatory effect by directly binding to C3 mRNA and inducing its expression [22]. Emerging evidence indicates that DDX5 regulates gene expression by participating in multiple RNA-related processes, including RNA processing, mRNA nuclear export, ribosome biogenesis, translation, and RNA decay [23]. Furthermore, DDX5 acts as a transcriptional coregulator that dynamically influences both the initiation and termination of transcription [24,25]. Elevated levels of DDX5 have been observed in human intestinal tumors, inflamed tissues from patients with IBD, and murine models of epithelial damage [26]. Recent studies revealed that DDX5 loss markedly protected against DSS-induced colitis and tumor formation in susceptible mice [22]. Thus, DDX5 represents a promising therapeutic target. Nevertheless, RBPs such as DDX5 have long been regarded as challenging drug targets because they lack well-defined, small-molecule binding pockets; there is high structural conservation across RNA binding domain family members; and much of their architecture is dominated by intrinsically disordered regions [27]. One therapeutic strategy beyond conventional protein-targeted approaches is to disrupt RBP function by precisely targeting their RNA binding interfaces to achieve selective inhibition while preventing complete protein ablation [28,29].

Magnesium isoglycyrrhizinate (MIG) is a clinically proven hepatoprotective drug widely used in clinical settings for managing acute/chronic hepatitis and chemotherapy-associated liver injury. Our previous work revealed that MIG could protect against DSS-induced colitis and maintain the intestinal barrier [30]. Here, by using limited proteolysis–mass spectrometry (LiP–MS) and thermal proteome profiling (TPP) chemoproteomic approaches, we identified MIG as a disruptor that specifically blocks DDX5-mediated β-catenin RNA binding. Moreover, we discovered that the DDX5 inhibitor MIG preserved intestinal barrier integrity following 5-FU-induced damage in vitro and in vivo. Mechanistically, MIG selectively inhibits the DDX5-mediated unwinding of the *CTNNB1* 5′ untranslated region (UTR) G-quadruplex (G4), induces β-catenin expression, improves intestinal barrier integrity, and reduces the severity of intestinal damage.

## Results

### Chemotherapy disrupts intestinal epithelial barrier integrity in patients with CRC

**Fig. 1.**
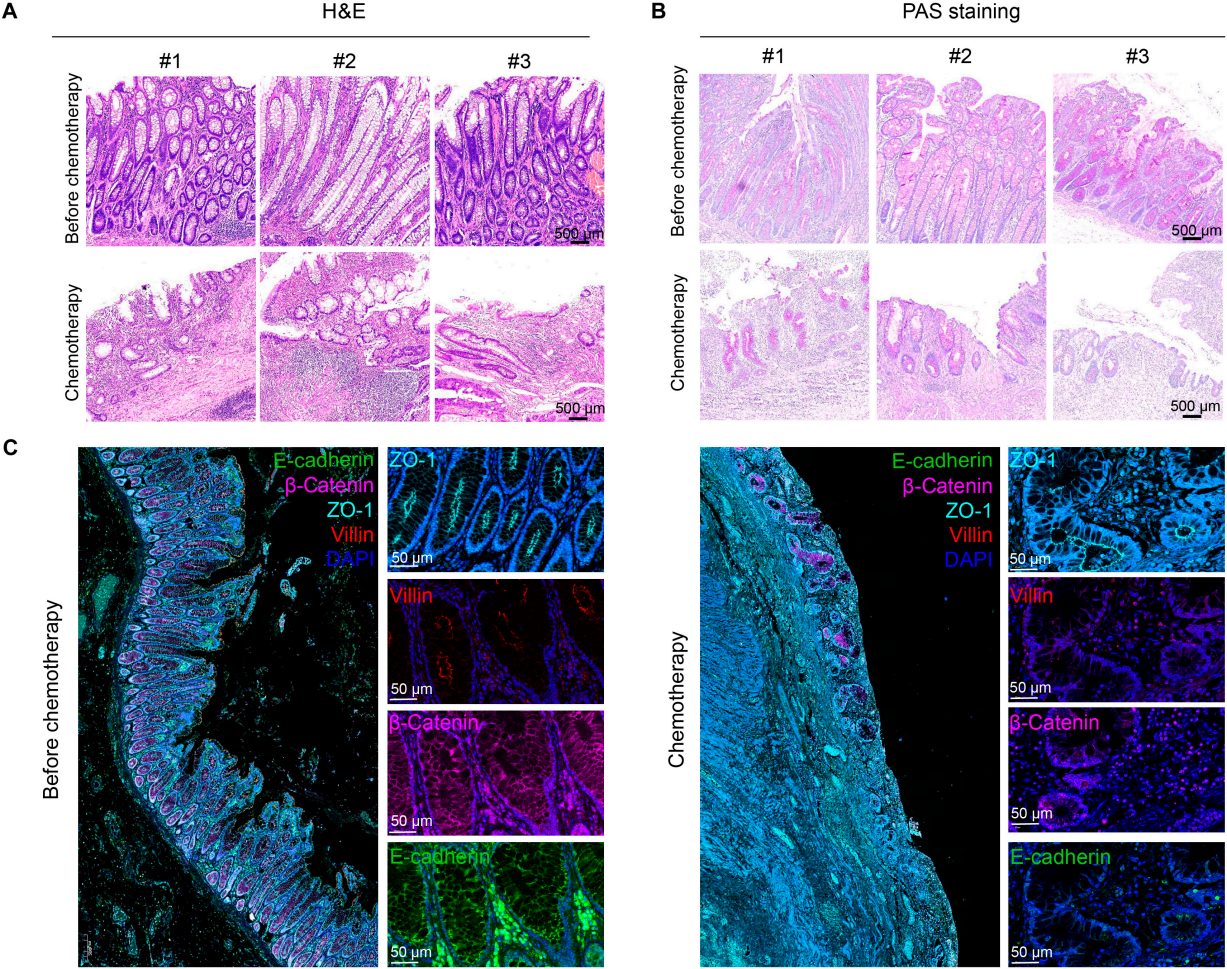
Chemotherapy disrupts the intestinal epithelial barrier in tumor-adjacent nontumor tissues in patients with CRC. (A) Hematoxylin and eosin (H&E)-stained images of colon tissues revealed exacerbated structural damage following chemotherapy. Scale bars, 500 μm. (B) PAS staining indicated reduced mucin production in colon tissues after chemotherapy. Scale bars, 500 μm. (C) Multiplex immunofluorescence analysis of the tight junction and adherens junction markers ZO-1 (blue), villin (red), β-catenin (purple), and E-cadherin (green) in adjacent intestinal tissues before and after chemotherapy. Scale bars, 50 μm.

Chemotherapy is widely used to treat metastatic CRC and gastric carcinoma. Chemotherapeutic agents disproportionately accumulate in intestinal crypts, increasing the risk of chemotherapy-induced intestinal damage compared with that in control tissues not exposed to chemotherapy. We analyzed tumor-adjacent nontumor intestinal tissues from 20 patients with CRC, including 10 who received chemotherapy and 10 who did not. Consistent with prior studies, chemotherapy led to a distorted crypt architecture, aggravated submucosal inflammation, and compromised barrier integrity (Fig. 1A and Fig. S1A). Furthermore, periodic acid–Schiff (PAS) staining revealed a pronounced loss of goblet cells following chemotherapy (Fig. 1B and Fig. S1B). Multiplex immunohistochemistry further demonstrated severe disruption of the intestinal epithelium, marked by a significant down-regulation of villin, ZO-1, E-cadherin, and β-catenin (Fig. 1C and Fig. S1C). Together, these results collectively demonstrate that chemotherapy undermines the structural and functional integrity of the intestinal epithelial barrier.

### MIG alleviates the intestinal damage induced by 5-FU and CPT-11

While we previously demonstrated that MIG protects mice from DSS-induced intestinal inflammation, these analyses did not explore its potential to ameliorate chemotherapy-induced intestinal damage. To address this, we utilized an MC-38 allograft model in which mice were treated with MIG (1.25 to 2.5 mg/kg, intraperitoneally) daily for 7 d following 5-FU administration. Intestinal damage was assessed via histopathological scoring and colon length measurements on day 7. We first evaluated whether MIG influences the antitumor efficacy of 5-FU. In vitro, MIG did not affect colon cancer cell survival. However, in vivo, compared with 5-FU monotherapy, the combination of MIG and 5-FU led to a decrease in both tumor weight and tumor volume (Fig. S2A to F), indicating that MIG enhances the therapeutic efficacy of 5-FU. We next examined the protective effects of MIG against 5-FU-induced intestinal injury. Cotreatment with MIG markedly improved the colon length and attenuated histopathological damage in mice (Fig. S2G). These improvements were characterized by reduced immune cell infiltration, decreased submucosal inflammation, and restoration of crypt architecture (Fig. S2H-I). PAS staining and quantitative polymerase chain reaction (qPCR) analysis further revealed that MIG counteracted the 5-FU-induced reduction in mucin production and junction-related genes (*Tjp1*, *Ocln*, *Cdh1*, and *Ctnnb1*) expression (Fig. S2J), supporting a role for MIG in preserving epithelial integrity.

**Fig. 2.**
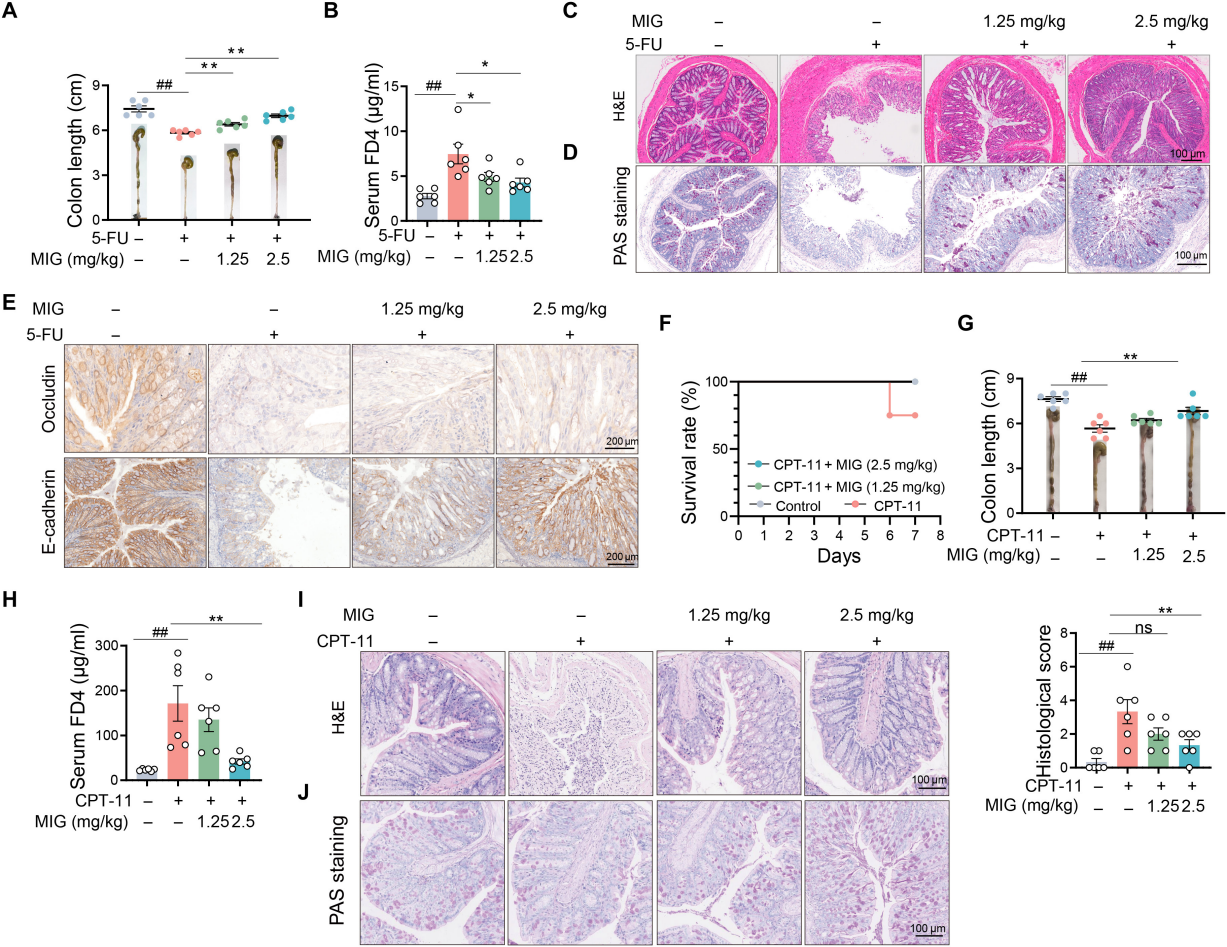
MIG attenuates intestinal injury induced by 5-FU or CPT-11. (A to E) MIG ameliorates 5-FU-induced intestinal injury. (A) Colon lengths and representative images. (B) Serum FD4 levels 4 h after oral administration on day 8. (C and D) Representative images of H&E and PAS staining of colon sections. Scale bars, 100 μm. (E) Immunohistochemical staining of the tight junction proteins occludin and E-cadherin. Scale bars, 200 μm. (F to J) MIG alleviates CPT-11-induced intestinal injury. (F) Survival curves. (G) Colon lengths and representative images. (H) Serum FD4 concentrations. (I and J) H&E-stained images, histological scores, and PAS-stained images of colon sections. Scale bars, 100 μm. The data are presented as the means ± SEMs (*n* = 6). Significance was determined by 2-tailed unpaired *t* tests or one-way ANOVA. ns, not significant. *P < 0.05 and ** and ##*P* < 0.01.

To validate these findings, we challenged mice with 5-FU for 7 d, which resulted in significant weight loss and colon damage. MIG cotreatment significantly mitigated these effects, reducing weight loss (Fig. S3A) and alleviating colon shortening (Fig. 2A). The fluorescein isothiocyanate (FITC)–dextran 4 (FD4) assay confirmed that MIG administration attenuated 5-FU-induced gut permeability in a dose-dependent manner (Fig. 2B), corroborating the protective effect of MIG, and preserved goblet cells (Fig. 2C and D and Fig. S3B and C). Immunohistochemistry also revealed increased expression of key junction proteins following cotreatment (Fig. 2E and Fig. S3D). Collectively, these results indicate that MIG effectively alleviates 5-FU-induced intestinal barrier damage without compromising its antitumor efficacy. We also established a preclinical model using irinotecan (CPT-11), a topoisomerase I inhibitor approved for metastatic CRC therapy following 5-FU treatment that elicits dose-limiting gastrointestinal damage in mice [31]. Administration of MIG (1.25 to 2.5 mg/kg) following a single dose of CPT-11 attenuated the resulting weight loss in a dose-dependent manner (Fig. S3E). MIG also significantly reduced lethality and ameliorated colon shortening at 2 different doses (Fig. 2F and G). Moreover, MIG markedly protected against intestinal barrier disruption, as evidenced by reduced permeability, preserved crypt architecture, and attenuated tissue damage (Fig. 2H to J and Fig. S3F), demonstrating its efficacy in ameliorating CPT-11-induced intestinal toxicity. Overall, these results confirmed that MIG mitigates intestinal toxicity induced by 5-FU and CPT-11 chemotherapy.

### MIG improves intestinal barrier function by maintaining cell junctions

**Fig. 3.**
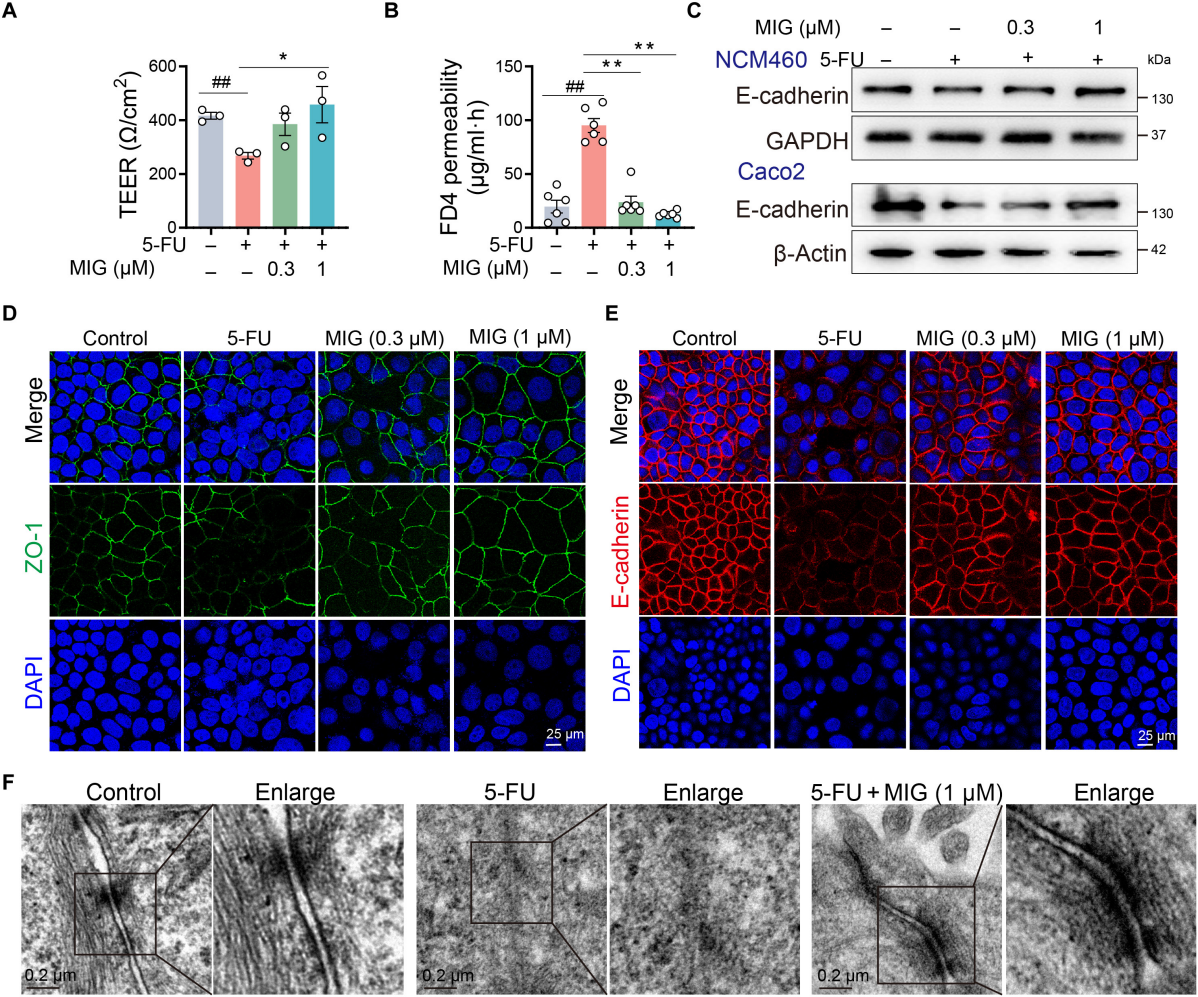
MIG decreases intestinal epithelial permeability and preserves intercellular junction protein expression. (A) TEER of Caco-2 cells following 24 h of treatment with 5-FU alone or in combination with MIG. (B) Epithelial permeability assessed by FD4 flux in Caco-2 cells treated as described in (A). (C) Western blot analysis of E-cadherin expression in Caco-2 and NCM460 cells after 24 h of treatment with 5-FU ± MIG. GAPDH, glyceraldehyde-3-phosphate dehydrogenase. (D and E) Immunofluorescence staining of E-cadherin (red) and ZO-1 (green) in Caco-2 cells treated with 5-FU and/or MIG for 24 h. DAPI, 4′,6-diamidino-2-phenylindole. (F) Representative transmission electron microscopy images of tight junctions in Caco-2 cells treated with 5-FU with or without MIG (1 μM). The data are presented as the means ± SEMs; *n* = 3 independent experiments. Significance was determined by a 2-tailed unpaired *t* test. *P < 0.05 and ** and ##*P* < 0.01.

To evaluate the protective effect of MIG on the intestinal epithelial barrier, we used an in vitro model in which IECs were used (Fig. S4A and B). 5-FU treatment significantly compromised barrier integrity, as indicated by decreased transepithelial electrical resistance (TEER) and increased FD4 permeability. Notably, cotreatment with 1 μM MIG effectively mitigated both of these effects (Fig. 3A and B). Western blot and immunofluorescence analyses revealed that 5-FU down-regulated key junction proteins, including E-cadherin and ZO-1, and disrupted their cellular distribution. These effects were reversed upon the addition of MIG, which restored both the expression and localization of these proteins (Fig. 3C to E and Fig. S4C and D). Furthermore, electron microscopy confirmed that MIG preserved the ultrastructural integrity of intercellular junctions following damage induced by 5-FU (Fig. 3F). Together, these results demonstrate that MIG reinforces intestinal barrier function by restoring the expression and organization of junctional proteins.

### MIG binds directly to the DDX5 protein

**Fig. 4.**
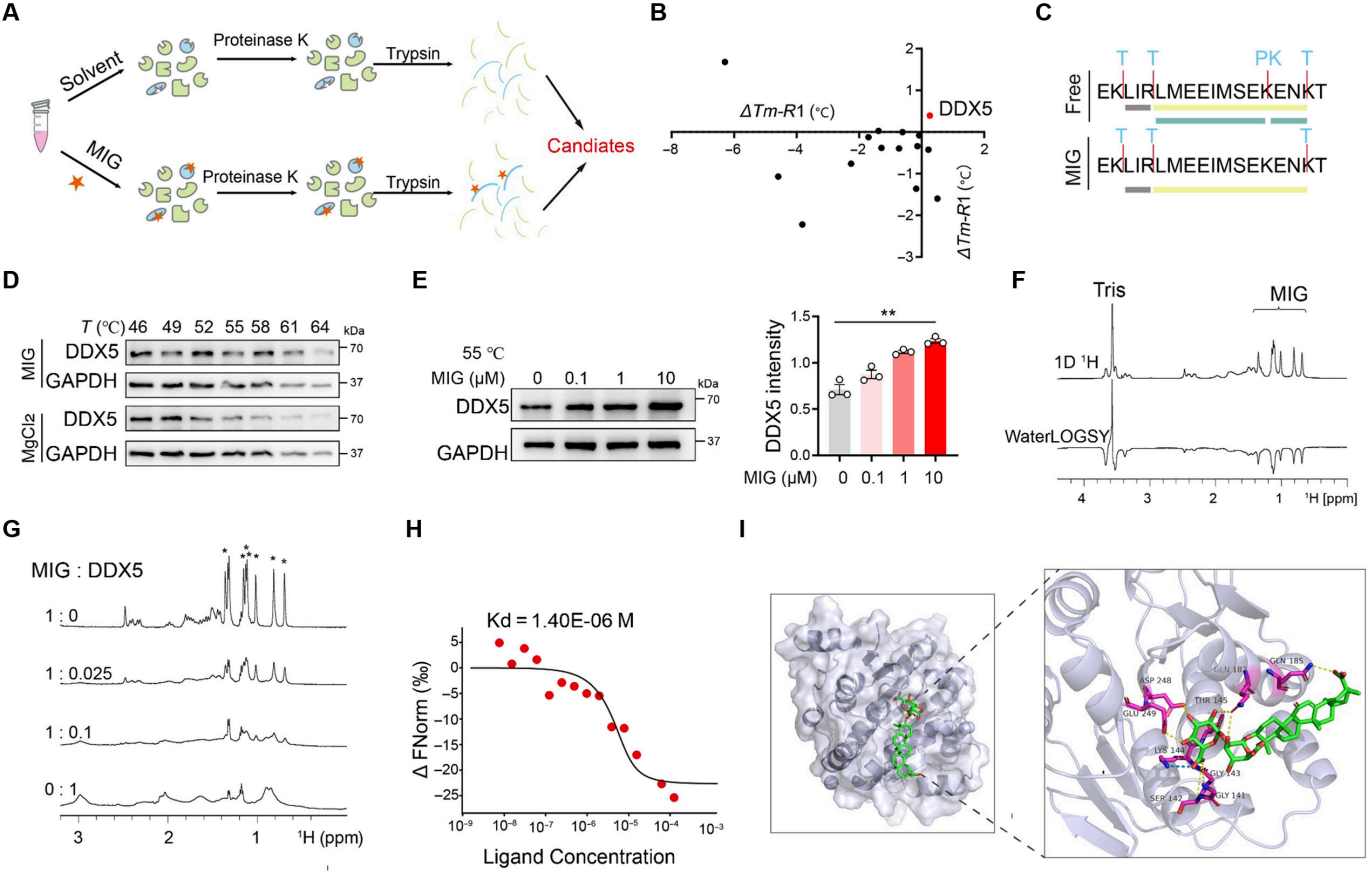
MIG directly binds to DDX5. (A) Experimental workflow for target engagement profiling using LiP–MS. (B) TPP-based identification of shifts in thermal stability upon MIG treatment. (C) LiP–MS analysis of DDX5 ± MIG. (D) CETSA data showing the thermal stabilization of DDX5 in Caco-2 cells treated with 1 μM MIG versus control treatment (MgCl_2_). (E) Dose-dependent stabilization of DDX5 by MIG (0 to 10 μM) assessed by CETSAs at 55 °C. (F) ^1^H NMR and WaterLOGSY spectra of 1 mM MIG with 10 μM DDX5. The peaks corresponding to tris and MIG are indicated. 1D, one-dimensional. (G) ^1^H NMR titration of 0.2 mM MIG with 0 to 0.1 equiv. of DDX5 in 20 mM tris-HCl (pH 7.5) and 100 mM NaCl at 310 K. The methyl peaks of MIG are marked with asterisks. (H) Binding affinity measured by MST, yielding a dissociation constant (*K*_d_) of 1.40 μM. (I) Molecular docking model of MIG bound to DDX5, with key interacting residues labeled. The data are shown as the means ± SEMs; *n* = 3 independent experiments. Significance was determined by a 2-tailed unpaired *t* test.**P < 0.01.

To elucidate the molecular mechanisms by which MIG maintains intestinal epithelial barrier integrity, we used LiP–MS to probe the direct binding targets of MIG in colonic epithelial cells (Fig. 4A and Table S1) and identified 14 candidate MIG-binding proteins. To validate these hits, we performed TPP, which confirmed that the thermal stability of DDX5—an RBP previously implicated in intestinal inflammation—increased upon MIG binding (Fig. 4B and C and Table S2). This interaction was further verified by a cellular thermal shift assay (CETSA): While DDX5 underwent significant degradation at 55 °C in control samples, MIG treatment stabilized the protein in a dose-dependent manner (Fig. 4D and E and Fig. S5A). We next purified recombinant DDX5 and probed its interaction with MIG via nuclear magnetic resonance (NMR). Chemical shift perturbations confirmed reversible binding between MIG and DDX5 (Fig. 4F and G and Fig. S5B). Further characterization by microscale thermophoresis (MST) revealed a dissociation constant (*K*_d_) of 1.40 μM, indicating the binding affinity (Fig. 4H). Computational docking suggested that MIG binds noncovalently to a surface pocket on DDX5 via residues Gly141, Ser142, Gly143, Lys144, Thr145, Val166, Gln182, Gln185, Asp248, and Glu249, potentially stabilizing the protein–ligand complex (Fig. 4I). Collectively, these results demonstrate that MIG selectively binds to DDX5.

### DDX5 unwinds the G4 of the *CTNNB1* 5′-UTR to inhibit its expression

**Fig. 5.**
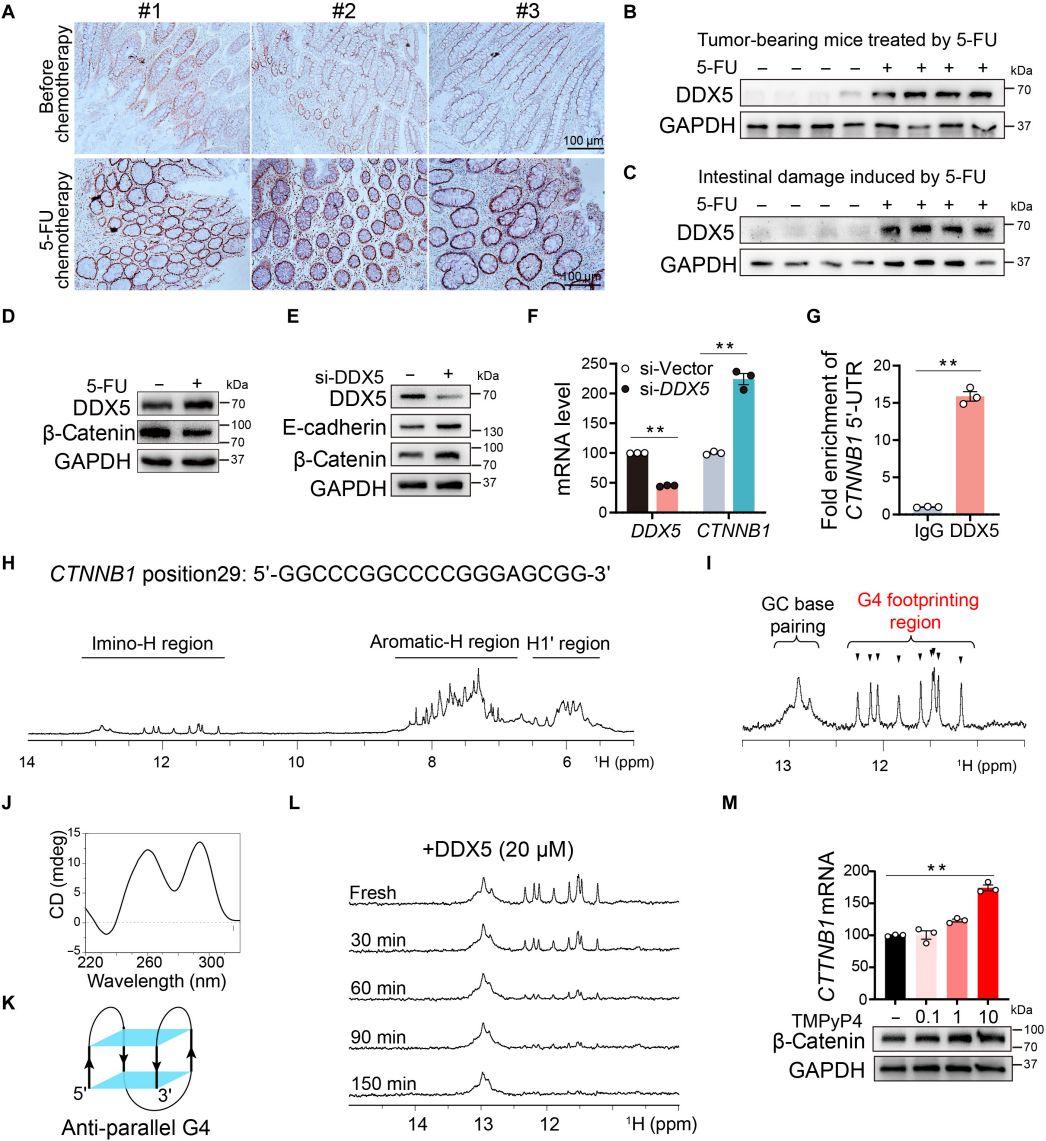
DDX5 is up-regulated during intestinal injury and resolves *CTNNB1* G4 structures.(A) DDX5 immunohistochemistry of tumor-adjacent nontumor tissues from patients with CRC. Scale bars, 100 μm. (B and C) Western blotting of DDX5 expression in colon tissues from (B) tumor-bearing and (C) nontumor-bearing mice after chemotherapy. (D) DDX5 and β-catenin expression in 5-FU-treated Caco-2 cells. (E and F) Protein (E) and mRNA (F) levels of DDX5 and β-catenin in Caco-2 cells transfected with si-*DDX5*. (G) ChIP with an anti-DDX5 antibody followed by qPCR of the *CTNNB1* 5′-UTR (*n* = 3). IgG, immunoglobulin G. (H) ^1^H NMR spectrum of the oligonucleotide at position 29. Imino, aromatic, and H1′ proton regions are indicated. (I) Imino proton region showing the coexistence of duplex and G4 structures in the presence of K^+^. (J) CD spectrum of position 29 in 100 mM KCl. (K) Topology of an antiparallel G4 formed by position 29 of the *CTNNB1* 5′-UTR. The strand direction (5′→3′) and G4 plane are highlighted. (L) NMR-based helicase assay: DDX5 (20 μM) disrupts G4 signals (11 to 12.5 ppm) while sparing duplex signals (12.5 to 13.5 ppm). (M) G4 stabilization by TMPyP4 (0 to 10 μM) increases β-catenin expression in Caco-2 cells. The data represent the means ± SEMs from 3 independent experiments. Statistical significance was determined by a 2-tailed unpaired *t* test. **P < 0.01.

To investigate the role of DDX5 in 5-FU-induced colonic injury, we first assessed its expression in clinical and preclinical models. Immunohistochemistry revealed higher DDX5 protein levels in patients with colon cancer who received 5-FU chemotherapy than in untreated patients (Fig. 5A, Fig. S6A). This increase was corroborated in colonic tissues from 5-FU-treated mice (Fig. 5B and C and Fig. S6B and C) and in Caco-2 cells stimulated with 5-FU (Fig. 5D and Fig. S6C). Consistent with previous observations, 5-FU also reduced the expression of junctional proteins, including E-cadherin and β-catenin (Fig. 5D and E, Fig. S6D, Fig. 3C). To determine whether DDX5 contributes to intestinal barrier dysfunction, we generated DDX5-knockdown Caco-2 cells. Silencing DDX5 increased the expression of E-cadherin and β-catenin (Fig. 5E and Fig. S6E)-*Ddx5*, suggesting an inverse correlation between DDX5 levels and intestinal barrier integrity.

Notably, DDX5 knockdown increased both β-catenin protein and *CTNNB1* mRNA levels (Fig. 5F), indicating transcriptional regulation. Previous work suggested that DDX5 binds to the *Ctnnb1* 5′-UTR in the mouse colonic epithelium [22]. Using chromatin immunoprecipitation (ChIP), we confirmed that DDX5 directly binds the *CTNNB1* 5′-UTR in human colonic epithelial cells (Fig. 5G). Given that DDX5 can unfold both RNA and DNA G4s in an adenosine triphosphate (ATP)-independent manner [32,33], we hypothesized that DDX5 regulates *CTNNB1* via G4 resolution.

 Quadruplex forming G-rich sequences (QGRS) mapper analysis revealed 3 putative G4-forming sequences in the GC-rich proximal region (where GC is guanine–cytosine base pair; position 29, GGCCCGGCCCCGGGAGCGG; position 57, GGAGGCGGAGACGGAGGAAGG; position 118, GGTCGAGGACGGTCGG) of the *CTNNB1* 5′-UTR (Fig. S6F). Using NMR, we found that only position 29 (29 nt upstream of the *CTNNB1* start site) formed a stable G4 structure in potassium buffer. Imino proton signals between 11 and 12.5 parts per million (ppm) and a positive circular dichroism (CD) peak at 295 nm indicated the formation of an antiparallel G4 topology (Fig. 5H and I). Thermal stability assays confirmed that this structure persists at physiological temperature (310 K; Fig. S6G). We next evaluated the ability of DDX5 to unwind this G4 structure. Using NMR under ATP-free conditions, we observed that DDX5 disrupted the *CTNNB1* G4 but not its duplex DNA (Fig. 5I, J, K, and L). Thus, we hypothesized that DDX5 regulates *CTNNB1* expression through a G4-dependent mechanism. To test this hypothesis, we used NMR to assess the ability of DDX5 to unwind the *CTNNB1* G4 structure in the absence of ATP. Consistent with our hypothesis, DDX5 unwound the *CTNNB1* G4 structure but failed to unwind DNA duplex structures (Fig. 5I and L). To assess the functional relevance, we treated Caco-2 cells with tetrakis(*N*-methyl-4-pyridyl)porphyrin (TMPyP4), a G4-stabilizing ligand [34]. TMPyP4 dose-dependently increased both *CTNNB1* mRNA and protein levels (Fig. 5M and Fig. S6H), supporting the role of G4 unfolding in DDX5-mediated repression. Together, these results demonstrate that DDX5 binds to the *CTNNB1* 5′-UTR and disrupts the regulatory G4 structure, leading to transcriptional repression.

### MIG specifically inhibits the DDX5-mediated unwinding of the *CTNNB1* G4

**Fig. 6.**
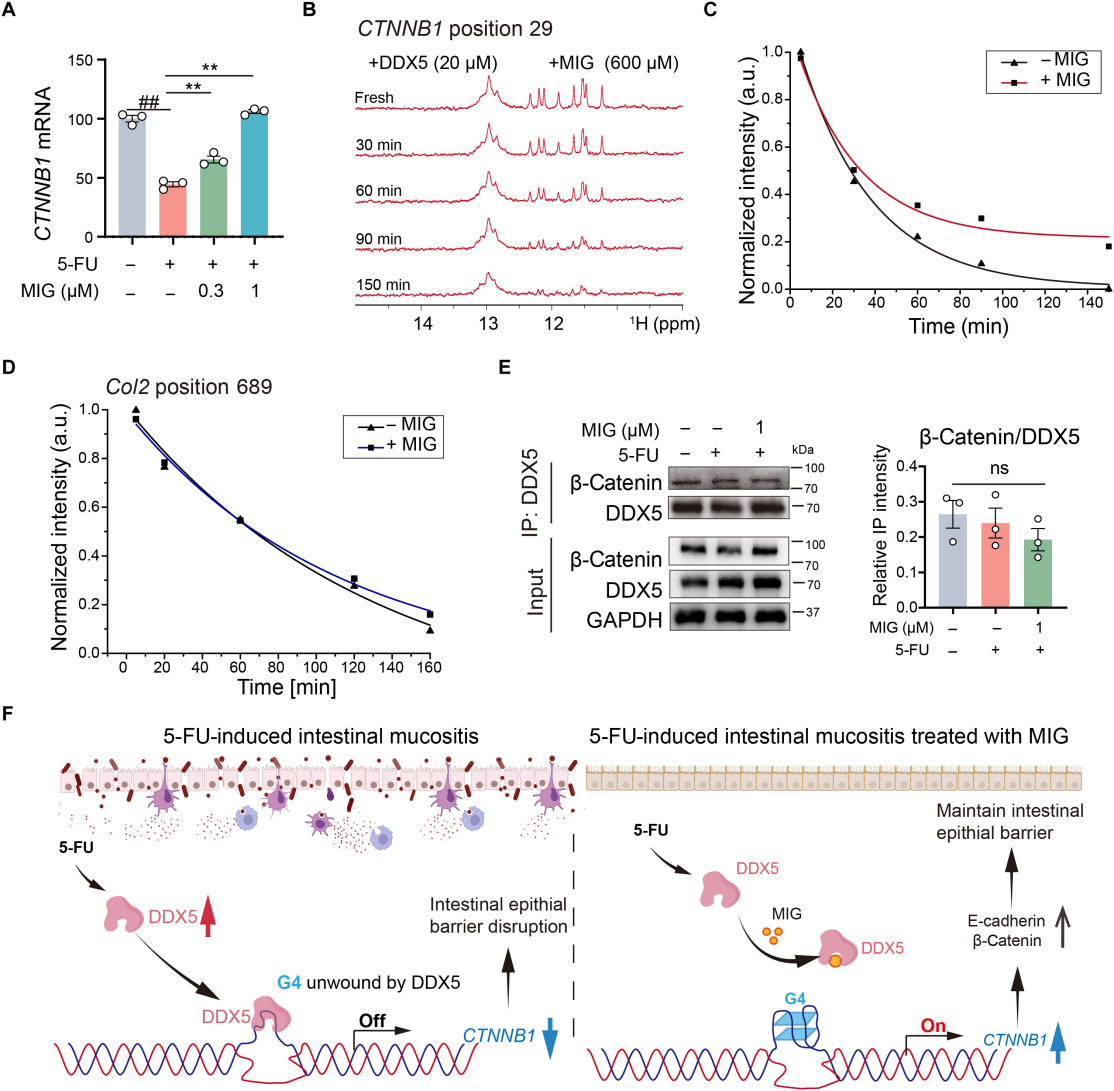
MIG specifically inhibits the *CTNNB1* unwinding effect of DDX5. (A) qPCR analysis of *CTNNB1* expression in Caco-2 cells after 24 h of MIG treatment (*n* = 3). (B and C) NMR helicase assays revealed that MIG (600 μM) impeded DDX5-induced unwinding of the G4 (imino proton region: 11.0 to 12.5 ppm). a.u., arbitrary units. (D) MIG (600 μM) did not affect the DDX5-mediated unwinding of the control Col2 G4 structure. (E) Coimmunoprecipitation (IP) and quantitative analysis of the DDX5–β-catenin interaction (*n* = 3). (F) Proposed mechanism through which MIG maintains intestinal epithelial barrier integrity. The data are presented as the means ± SEMs; statistical significance was determined by a 2-tailed unpaired *t* test. ** and ##*P* < 0.01.

Exposure to 5-FU reduced *CTNNB1* transcript levels in Caco-2 cells, whereas cotreatment with MIG restored *CTNNB1* expression in a dose-dependent manner (Fig. 6A). To assess whether MIG impairs the ability of DDX5 to resolve G4 structures, we performed real-time ^1^H NMR under near-physiological conditions (100 mM KCl, 310 K, pH 6.8). The imino proton signals (11.5 to 12.5 ppm) corresponding to the G4 structure formed by position 29 persisted significantly longer in the presence of MIG (600 μM) than in its absence, indicating that MIG attenuates the *CTNNB1* G4 unwinding activity of DDX5 (Fig. 5L and 6B and C). To evaluate the selectivity of MIG, we examined its effect on the DDX5-mediated unwinding of a G4 structure within the *Col2 *promoter. DDX5 exhibited inherently weaker resolvase activity toward *Col2* than toward *CTNNB1*. Notably, MIG did not alter the efficiency of DDX5 in unwinding the *Col2* G4 (Fig. 6D and Fig. S7A and B), underscoring its selective inhibition of *CTNNB1*. Since DDX5 has been shown to interact with transcription factors such as β-catenin in human cancer cells [35,36], we investigated whether MIG influences this interaction. Coimmunoprecipitation assays revealed that MIG had no significant effect on the binding of DDX5 to β-catenin (Fig. 6E and F). Together, these findings demonstrate that MIG specifically inhibits the G4 unwinding activity of DDX5 at the *CTNNB1* locus without broadly disrupting its protein–protein interactions.

### MIG sustains intestinal barrier function via DDX5 blockade

**Fig. 7.**
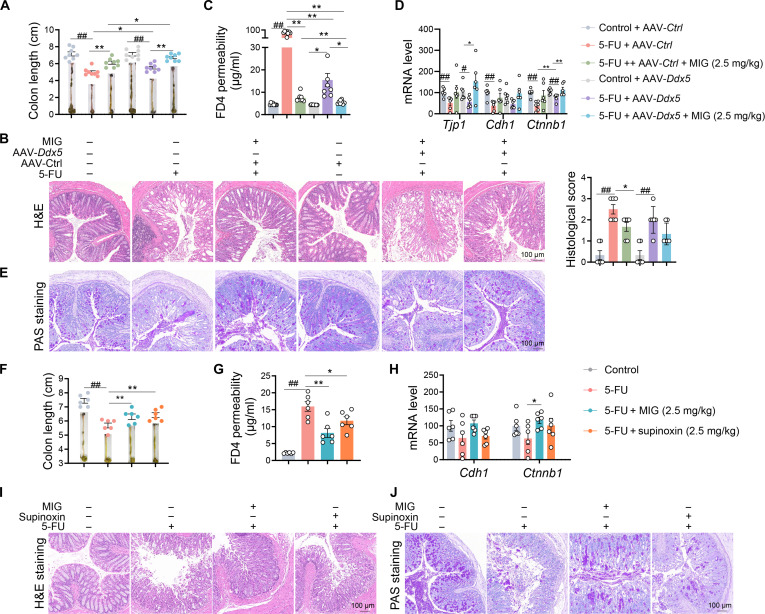
MIG ameliorates 5-FU-induced intestinal injury to an extent comparable to genetic *Ddx5* knockdown and pharmacological DDX5 inhibition. (A to E) Mice (*n* = 6 to 8) were administered AAV-*Ddx5* or AAV-Ctrl (1.5 × 10^11^ GC/ml) via the rectum. After 7 d, the mice were treated with 5-FU (50 mg/kg, intraperitoneally) and MIG (2.5 mg/kg, intraperitoneally) for 7 d. (A) Colon lengths. (B) H&E-stained images of colon sections. Scale bar, 100 μm. (C) Intestinal permeability as determined by serum FITC–dextran levels. (D) qPCR analysis of ZO-1, E-cadherin, and β-catenin mRNA expression. (E) Images of PAS staining. Scale bar, 100 μm. (F to J) Mice (*n* = 6) were treated with 5-FU (50 mg/kg, intraperitoneally), followed by MIG (2.5 mg/kg, intraperitoneally) or supinoxin (2.5 mg/kg, intraperitoneally) for 7 d. (F) Colon lengths. (G) Serum FITC–dextran levels. (H) qPCR analysis of *Cdh1* and *Ctnnb1* mRNA levels. (I) H&E-stained images. Scale bar, 100 μm. (J) Images of PAS staining. Scale bar, 100 μm. The data are presented as the means ± SEMs; *n* = 6. Significance was determined by 2-tailed unpaired *t* tests or one-way ANOVA.*P < 0.05 and ** and ##*P* < 0.01.

Given the central role of DDX5 in intestinal epithelial barrier integrity, we evaluated the therapeutic benefit of intestinal epithelium-specific *Ddx5* knockdown, both alone and in conjunction with MIG (2.5 mg/kg), in a murine model of 5-FU-induced damage. Compared with control mice, mice subjected to *Ddx5* knockdown—with or without MIG coadministration—exhibited significantly attenuated weight loss (Fig. S8A and B). Furthermore, compared with that in 5-FU-treated mice, the colon lengths in *Ddx5*-deficient mice were notably preserved, and histopathological injury was reduced. The combination of *Ddx5* knockdown and MIG had additive protective effects, including further increases in colon length and enhancements in crypt architectural conservation (Fig. 7A and B). Compared with the control group, the combination group also exhibited superior barrier function, as indicated by reduced serum FD4 levels, elevated goblet cell numbers, and increased expression of junctional proteins (Fig. 7C to E, Fig. S8C).

We next compared MIG with supinoxin (RX-5902), a known DDX5 inhibitor that binds phospho-Tyr593 and attenuates β-catenin nuclear accumulation [37]. While supinoxin (2.5 mg/kg) outperformed MIG at the same dose in ameliorating gross toxicity, such as weight loss and colon shortening (Fig. 7F and Fig. S8D), MIG was notably more effective at mitigating microscopic and functional damage, conferring greater histopathological protection, reduced intestinal permeability, increased goblet cell abundance, and robustly increased junction protein expression (Fig. 7G-J and Fig. S8E and F). Collectively, these data underscore that targeted inhibition of DDX5 with MIG substantially protects against 5-FU-induced compromise of the intestinal epithelial barrier.

## Discussion

Chemotherapy-induced intestinal damage is a complex condition with incompletely elucidated mechanisms. While extensive research has established immune cell dysregulation as a critical driver of injury, emerging evidence highlights the intestinal epithelium as a pivotal site of pathogenesis [38]. However, elucidating the pathogenic mechanisms of intestinal epithelial barrier damage is challenging. Here, we used MIG (which has been suggested to maintain intestinal epithelial barrier function) as a probe to explore the underlying mechanism. Our study revealed that the up-regulation of DDX5 induced by 5-FU treatment directly destabilizes the G4 structure in the 5′-UTR of *CTNNB1* (β-catenin), reducing its expression. Next, we identified MIG as a first-in-class, highly selective inhibitor of DDX5-mediated G4 unwinding. Crucially, MIG directly stabilizes the G4 structure within the 5′-UTR of the β-catenin-encoding gene *CTNNB1*, thereby promoting its transcription and consequently maintaining β-catenin protein levels. This molecular intervention restores intestinal barrier integrity and mitigates 5-FU-induced gastrointestinal toxicity. Our preclinical data demonstrated that MIG confers significant protection against 5-FU-induced intestinal injury through β-catenin-mediated barrier preservation. Notably, our findings suggest that the therapeutic potential of MIG may extend beyond chemotherapy-induced intestinal damage to other barrier dysfunction-related pathologies.

Despite growing research interest in intestinal epithelial barrier repair, no clinical drugs targeting barrier components (particularly cell junctions) are yet available. Traditional Chinese medicine holds unique therapeutic potential for addressing intestinal barrier dysfunction and the associated diarrhea. This is exemplified by PHY906, a formulation derived from Huang Qin Tang, which has been shown to ameliorate CPT-11-induced gastrointestinal toxicity in phase I/II clinical trials [39]. Our research revealed that MIG, a fourth-generation optimized derivative of glycyrrhizic acid (a key bioactive constituent of PHY906), had the expected mucosal protective effect on 5-FU/CPT-11-induced gastrointestinal toxicity. In addition, our findings demonstrated that MIG treatment mitigated chemotherapy-mediated toxicity while preserving antitumor activity. One possible explanation for these results is that the MIG-mediated increase in β-catenin expression has anticancer effects and does not increase CRC risk, likely because of its compartmentalized regulation of β-catenin. In addition, our unpublished MS data revealed that MIG primarily modulates the adherens junction pathway, increasing membrane-associated β-catenin expression but not its nuclear accumulation. Intriguingly, pharmacological intervention with MIG resulted in superior protection of the barrier against 5-FU-induced intestinal damage compared with genetic *Ddx5* knockdown or DDX5 inhibitor treatment. Furthermore, combining *Ddx5* knockdown with MIG resulted in enhanced protective effects. A plausible explanation for these results is that the residually expressed DDX5 protein following knockdown may retain partial G4 *CTNNB1* mRNA unwinding activity. MIG likely inhibits this remaining activity, thereby augmenting the recovery of β-catenin mRNA expression. Our data suggest that MIG could be a promising therapeutic to address the critical need for agents capable of protecting intestinal barrier function without compromising anticancer treatment efficacy. Owing to its unique ability to regulate β-catenin localization, MIG is an ideal molecular probe for investigating the fundamental biological mechanisms of epithelial barrier maintenance and cancer pathogenesis.

DDX5, identified as a direct MIG-interacting protein, has tissue protective effects in certain contexts [32,40], yet its role in intestinal pathology appears to be context dependent. Indeed, our data suggest that the up-regulation of DDX5 induced by 5-FU significantly impaired intestinal epithelial barrier integrity and particularly affected cell junctions. This increase in DDX5 expression is correlated with marked reductions in both β-catenin mRNA and protein levels through a mechanism distinct from its canonical transcriptional coactivator function. These findings establish that DDX5 functions as a novel negative regulator of β-catenin via a noncanonical RNA-mediated regulatory pathway in chemotherapy-induced epithelial injury. Our prior work revealed that DDX5 possesses G4 resolving activity that is independent of its helicase activity. In this study, we revealed that DDX5 binds to the *CTNNB1* 5′-UTR and that its stabilization of the G4 structure leads to the up-regulation of β-catenin, counteracting the corresponding suppressive effect of DDX5. Using NMR, we determined that the 5′-UTR of the *CTNNB1* G4 was resolved by DDX5 under ATP-free conditions. Collectively, these findings redefine the pathogenic role of DDX5 in intestinal physiology. In addition, this mechanism suggests that DDX5 is a promising therapeutic target for 5-FU-induced intestinal barrier dysfunction. Given the multiple beneficial roles of MIG in other tissues, we infer that colon-targeted delivery could amplify the therapeutic benefits of MIG while minimizing side effects to treat intestinal damage. Thus, this target-specific intervention strategy will guide our subsequent lead optimization efforts.

Notably, our research has several limitations. For example, we did not investigate the mechanism by which 5-FU increases the expression of DDX5. In addition, we did not characterize whether MIG stabilized G4 structures within other genes. Recent studies, including this one, have revealed that DDX5 unwinds the G4 structure of multiple genes, such as *Myc*, *Col2*, and *CTNNB1*, but how DDX5 discriminates between targets remains unclear; thus, further investigations are needed to clarify how DDX5 recognizes specific sequences.

This study provides a rationale for the further exploration of the intestinal epithelial barrier as a therapeutic target for mitigating chemotherapy-induced injury. Here, we present compelling preclinical evidence that MIG confers significant protection against 5-FU-induced gastrointestinal toxicity through the preservation of intestinal epithelial barrier function. The therapeutic potential of MIG may extend beyond chemotherapy-induced intestinal damage to other barrier dysfunction-related pathologies, including cancer, IBD/Crohn’s disease, and intestinal infections.

## Materials and Methods

### Subjects

We studied paraffin-embedded colon tissue samples from patients with CRC who had received 5-FU treatment (*n* = 10) or no chemotherapy (*n* = 10) collected from the Pathology Department of Jiangsu Provincial Hospital of Traditional Chinese Medicine (approval number: 2023NL-002-02).

### Cell Counting Kit-8 assay

Cell Counting Kit-8 assays were carried out according to established methods [41].

### Mouse model

Three independent mouse models were used.

#### Syngeneic tumor model

MC38 cells (2 × 10^6^) were implanted subcutaneously into the right flank of each mouse. When the tumor volume reached ≈100 mm^3^, the mice were randomized into 4 groups (*n* = 6 per group): group I, vehicle (normal saline, intraperitoneally, daily); group II, 5-FU (50 mg/kg, intraperitoneally, days 1 to 7); group III, 5-FU + MIG (1.25 mg/kg, intraperitoneally, days 1 to 7); group IV, 5-FU + MIG (2.5 mg/kg, intraperitoneally, days 1 to 7). The tumor dimensions (length [*L*] and width [*W*]) were measured periodically, and the tumor volume was calculated as *V* = *L* × *W*^2^/2. On day 8, all mice were euthanized, and the tumors were excised, weighed, and processed for further analysis.

#### Nontumor-bearing mouse model

C57BL/6J mice were assigned to 4 groups (*n* = 6 per group) and received the following treatments: group I, vehicle (saline, intraperitoneally); group II, 5-FU (50 mg/kg, intraperitoneally, days 1 to 7); group III, 5-FU + MIG (1.25 mg/kg, intraperitoneally, days 1 to 7); group IV, 5-FU + MIG (2.5 mg/kg, intraperitoneally, days 1 to 7). Body weight was recorded daily. All mice were euthanized on day 8, and intestinal tissues were collected for histopathological analysis.

#### Supinoxin treatment model

Male C57BL/6J mice (20 to 22 g) were randomly divided into 4 groups (*n* = 6 per group) and treated as follows: group I, normal saline (intraperitoneally, daily); group II, 5-FU (50 mg/kg, intraperitoneally, days 1 to 7); group III, 5-FU + MIG (2.5 mg/kg, intraperitoneally, days 1 to 7); group IV, 5-FU + supinoxin (2.5 mg/kg, intraperitoneally, days 1 to 7). The mice were euthanized 7 d after the final treatment.

### Intestinal permeability measurements

In vivo, after a 4-h fast, the mice were orally administered 200 μl of FD4 (60 mg/ml, 600 mg/kg) via gavage [19]. Blood samples were collected 4 h postadministration, and serum FITC–dextran levels were quantified using a fluorescence microplate reader (excitation, 485 nm; emission, 535 nm). In vitro, Caco-2 cells were cultured on Transwell dishes for 18 d until they formed confluent monolayers, and the medium was changed every 48 h. FD4 (1 mg/ml) was applied to the apical compartment. After 1 h of incubation at 37 °C, the fluorescence intensity in the basolateral medium was measured in duplicate using a microplate reader. The TEER was monitored daily until the values plateaued.

### Western blotting and coimmunoprecipitation

We performed Western blotting and coimmunoprecipitation as described previously [42].

### Immunohistochemistry and immunofluorescence

We performed immunohistochemistry and immunofluorescence as described previously [43].

### NMR spectroscopy

The oligonucleotide concentrations of the samples were quantified by measuring the ultraviolet absorbance of each sample at 260 nm using a One Drop OD-1000 Plus spectrophotometer. The NMR samples were dialyzed against ultrapure water prior to analysis to minimize background signal interference and enhance the quality of the spectra. NMR spectra were acquired using a 500-MHz Bruker spectrometer equipped with a cryogenically cooled probe at temperatures ranging from 288 to 315 K. Gradient-tailored excitation (WATERGATE) was used as a water suppression technique in the one-dimensional ^1^H NMR experiments on samples in 90% H_2_O/10% D_2_O. Water–ligand observed via gradient spectroscopy (WaterLOGSY) experiments were conducted to investigate the binding of the small molecule MIG to the DDX5. The experiment utilized the ephogsypgno 2-pulse sequence with a mixing time of 1,000 ms to allow magnetization transfer via spin diffusion among the water, protein, and ligand protons. In addition, the temperature was maintained at 310 K to mimic physiological conditions. The raw datasets were processed and analyzed using Bruker TopSpin software (version 3.6.2).

### Thermal proteome profiling

The TPP assay was conducted according to previously reported methods [44]*.* Briefly, protein samples isolated from Caco-2 cells were aliquoted and incubated with either MgCl_2_ (control) or MIG. Each sample was then divided into 8 aliquots, which were heated to different temperatures ranging from 37 to 67 °C. After digestion with trypsin, conventional data-dependent acquisition MS data were acquired and used to construct a spectral library of the proteins obtained from the samples treated at 37 °C. We subsequently used data-independent acquisition to collect MS data for each sample. On the basis of the protein quantification data, the melting curves of each protein were fitted using the Bioconductor TPP package, from which the melting points were calculated. The differences in the melting points of the proteins between the dimethyl sulfoxide (DMSO) group and the group treated at 5 °C were subsequently compared.

### Limited proteolysis–mass spectrometry

Caco-2 cells were incubated with or without 10 μM MIG for 24 h. LiP was then carried out using proteinase K under native conditions, followed by complete tryptic digestion under denaturing conditions to prepare peptides for MS analysis. [45]

### MST assay

The binding affinities of the compounds for DDX5 were measured using a Monolith NT.115 instrument (NanoTemper). DDX5 was fluorescently labeled with RED–tris–nitrilotriacetic acid (RED-NHS 2nd Generation kit) at a 1:3 protein:dye molar ratio, and the free dye was removed by desalting. For the assay, 16-point serial dilutions of each compound (from 100 μM) were mixed with 50 nM labeled DDX5 in assay buffer (50 mM tris-HCl [pH 7.4], 150 mM NaCl, and 0.05% Tween 20) containing a final concentration of 0.5% DMSO. Following a 10-min incubation at 25 °C, the samples were loaded into standard capillaries and measured at 40% light-emitting diode/medium MST power. Dissociation constants (*K*_d_ values) were determined by fitting the MST data to the *K*_d_ model in MO.Affinity Analysis software (NanoTemper), and curves were plotted in GraphPad Prism (v.8.02). [46]

### CD spectroscopy experiment

The sample, previously confirmed by NMR spectroscopy to exhibit the expected G4 structure, was diluted in buffer E (20 mM tris-HCl [pH 7.5] and 100 mM KCl) to a final concentration of 50 μM. CD spectra were acquired immediately at ambient temperature (∼25 °C) on a JASCO J-810 spectropolarimeter (Japan) fitted with a 0.1-cm path length quartz cuvette. Wavelength scans were performed from 220 to 320 nm at a rate of 100 nm/min. Each spectrum represents the average of 3 successive scans and was baseline-corrected against buffer E. Data were processed and analyzed using Spectra Manager software (v.2.15.01) [47].

### ChIP assay

ChIP was conducted according to previously reported methods [30].

### Statistics

All quantitative data were analyzed using GraphPad Prism software (v. 8.0.1; GraphPad Software Inc.). Specific statistical tests applied to the data from each experiment are detailed in the corresponding figure legends. In general, comparisons between 2 groups were conducted using 2-tailed unpaired *t* tests or Mann–Whitney *U* tests, comparisons among 3 or more groups were performed using one-way analysis of variance (ANOVA) with appropriate post hoc tests. Significance was defined at *P* < 0.05.

## Data Availability

All data associated with this study are present in the paper or the Supplementary Materials. Additional raw data for the experiment are also available upon request.
