# Supplementary material for: Inhibition of DDX5-Mediated G-Quadruplex Unwinding in the β-Catenin 5′-UTR by Magnesium Isoglycyrrhizinate Alleviates Chemotherapy-Induced Intestinal Injury
Source: Research (Wash D C). 2026 Mar 19;9:1044. doi: 10.34133/research.1044 (PMC13000115; doi:10.34133/research.1044)
Supplement: Supplementary 1 — Supplementary Methods Figs. S1 to S8 Tables S1 and S2 [file research.1044.f1.zip › supplemental doc file_research.1044_March 5 CLEAN.docx]

Supplementary Materials for

**Inhibition of DDX5-Mediated G-Quadruplex Unwinding in β-catenin 5'-UTR by Magnesium Isoglycyrrhizinate Alleviates Chemotherapy-Induced Intestinal Injury**

Materials and Methods

Figs. S1 to S8

Table S1 Lip-MS results

Table S2 TPP results

**SUPPLEMENTAL METHODS**

**Reagents:** 5-FU (molecular weight of 130.08) was purchased from Sigma‒Aldrich Corporation (St. Louis, MO). CPT-11 (molecular weight of 623.14) was purchased from Yuanye Bio-Technology Co., Ltd. (Shanghai, China). MIG was obtained from Chia Tai Tianqing Pharmaceutical Group Co., Ltd. (Nanjing, China; lot numbers: 215020, 150819104 and 150829204). The following antibodies were used in this study: anti-occludin (Santa Cruz, Dallas, TX, USA; sc-133256), anti-E-cadherin (BD Biosciences, Franklin Lakes, NJ, USA; 2045737), anti-ZO-1 (Cell Signaling Technology, Beverly, MA, USA; #13663), anti-β-catenin (BD Biosciences, Franklin Lakes, NJ, USA; 610153), anti-Ddx5 (Atlas Antibodies, Stockholm, Sweden; HPA020043), and anti-Ki67 (Proteintech, Wuhan, China; 27309-1-AP). A Cell Counting Kit 8 (CCK-8) was purchased from Vazyme Biotech Co., Ltd. (Nanjing, China). An immunohistochemical (IHC) analysis kit (3,3ʹ-diaminobenzidine (DAB)) was procured from Proteintech (Wuhan, China). FD4 (average molecular weight of 3000-5000) was obtained from Sigma‒Aldrich Corporation (St. Louis, MO, USA).

**Animals:** C57BL/6J mice (male, 6‒8 weeks of age, 22‒24 g) were purchased from GemPharmatech Co., Ltd. (Nanjing, Jiangsu, China). All mouse experiments were conducted following the Guide for the Care and Use of Laboratory Animals (Ministry of Science and Technology of China, 2006) and were approved by the Animal Ethics Committee of Nanjing University of Chinese Medicine (no. 202405A058). The mice were housed at the Experimental Animal Center of Nanjing University of Chinese Medicine at 23–26°C and 40–60% humidity for 7 days on a 12 h dark/night cycle with free access to food and water. All efforts were made to reduce the number of animals used and minimize animal suffering.

**Cell culture**: A human colon cancer cell line (Caco-2), a human normal colonic epithelial cell line (NCM460) and a mouse colon cancer cell line (MC-38) were obtained from the Type Culture Collection of the Chinese Academy of Sciences (Shanghai, China). Caco-2 and MC-38 cells were cultured in DMEM and NCM460 cells were cultured in 1640 medium. All media were supplemented with 1% penicillin/streptomycin, and the cells were cultured in a constant humidity incubator with 5% CO2 at 37°C.

**Quantitative real-time PCR (qRT-PCR):** The protocols for qRT-PCR has been described previously[1]. The primer sequences used in the study were described as follows:

*GAPDH*HomoFor: 5′-GTCTCCTCTGACTTCAACAGCG-3′;

*GAPDH*HomoRev: 5′-ACCACCCTGTTGCTGTAGCCAA-3′;

*CDH1*HomoFor: 5′-GCCTCCTGAAAAGAGAGTGGAAG′;

*CDH1*HomoRev: 5′-TGGCAGTGTCTCTCCAAATCCG-3′;

*CTNNB1*HomoFor: 5′-CACAAGCAGAGTGCTGAAGGTG′;

*CTNNB1*HomoRev: 5′-GATTCCTGAGAGTCCAAAGACAG-3′;

*Gapdh*MusFor: 5′-CATCACTGCCACCCAGAAGACTG-3′;

*Gapdh*MusRev: 5′-ATGCCAGTGAGCTTCCCGTTCA-3′;

*Ctnnb1*MusFor: 5′-GTTCGCCTTCATTATGGACTGCC′;

*Ctnnb1*MusRev: 5′-ATAGCACCCTGTTCCCGCAAAG′.

*Cldn1*MusFor: 5′-TGCCCCAGTGGAAGATTTACT-3′;

*Cldn1*MusRev: 5′-CTTTGCGAAACGCAGGACAT-3′;

*Tjp1*MusFor: 5′-GGGGCCTACACTGATCAAGA-3′;

*Tjp1*MusRev: 5′-TGGAGATGAGGCTTCTGCTT-3′;

*Ocln*MusFor: 5′-TTGAAAGTCCACCTCCTTACAGA-3′;

*Ocln*MusRev: 5′-CCGGATAAAAAGAGTACGCTGG-3′;

*Cdh1*MusFor: 5′-AATGAAGCCCCCATCTTTAT-3′;

*Cdh1*MusRev: 5′-GCGTCTTCTCTGTCCATCTC-3′.

**DDX5 protein purification:** The plasmid pMAL-MBP-DDX5-GST, obtained from General Biosystems, Inc. (China), was transfected into *Escherichia coli* Rosetta (DE3) competent cells via a heat shock method. Protein expression was induced by adding IPTG to a final concentration of 0.2 mM when the bacterial culture reached an OD_600_ of 0.6–0.8(*47*). The culture was then incubated at 16°C with shaking at 180 rpm for 20 h. The cells were harvested by centrifugation, resuspended in Buffer A (50 mM Tris-HCl, pH 8.0, 300 mM NaCl, 2 mM DTT, and 10% glycerol), and lysed via ultrasonication. The soluble lysate was subjected to affinity purification using amylose resin to capture the MBP-DDX5-GST fusion protein, which was subsequently eluted with Buffer B (50 mM Tris-HCl, pH 8.0, 300 mM NaCl, and 9.2 mM maltose). To further purify the protein, the eluate was applied to glutathione resin, and the target protein was eluted with Buffer C (50 mM Tris-HCl, pH 8.0, 300 mM NaCl, and 10 mM reduced glutathione). The purified protein was buffer-exchanged into Buffer D (50 mM Tris-HCl, pH 8.0, and 100 mM NaCl) and concentrated via centrifugal ultrafiltration. The protein concentration was determined by measuring the UV absorbance at 280 nm using a One Drop OD-1000 plus spectrophotometer.

**CETSA assay:** Caco-2 cells were treated with either MgCl_2_ or 10 μM MIG for 3 h. Subsequently, the cells were harvested and subjected to CETSA.

**Molecular docking:** The crystal structure of DDX5 (PDB ID: 3FE2) was downloaded from the Protein Data Bank (PDB) (https://www.rcsb.org) and preprocessed using the Protein Preparation Wizard module in Schrödinger Maestro 2019 by removing water molecules, adding polar H atoms, and assigning Gasteiger–Hückel charges. H-optimization (0.01 kcal/mol convergence, 1000 iterations) and OPLS3 refinement were performed, and the output was saved as a .pdb file. The ligand (IG) was constructed in ChemBio 3D Ultra and then processed with the LigPrep package of Schrödinger software using the OPLS 2005 force field. The docking score of MIG bound to the X-ray crystal structure of DDX5 was calculated using the docking module in Schrödinger Maestro 2019.

***Ddx5* knockdown *in vivo*:** shRNAs (si*Control*: 5´-UUCUCCGAACGUGUCACGUTT-3´; and sh*Ddx5*: 5´-CCGGCCTGGAAGACATTGACTTT-3´) were synthesized by PackGene (Guangzhou, China). C57BL/6J mice were rectally injected with AAV-*Ddx5* or AAV-*Ctrl* (1.5^E+11^ GC/mL), followed by intraperitoneal injection of 50 mg/kg 5-FU seven days later and intraperitoneal injection of MIG (2.5 mg/kg) for 7 days. Colon tissues were subsequently collected for further analysis.

[1] Cui, J., Li, Y., Jiao, C., Gao, J., He, Y., Nie, B., Kong, L., Guo, W. and Xu, Q. (2021) Improvement ofmagnesium isoglycyrrhizinate on DSS-induced acute and chronic colitis. Int Immunopharmacol, 90, 107194.

**
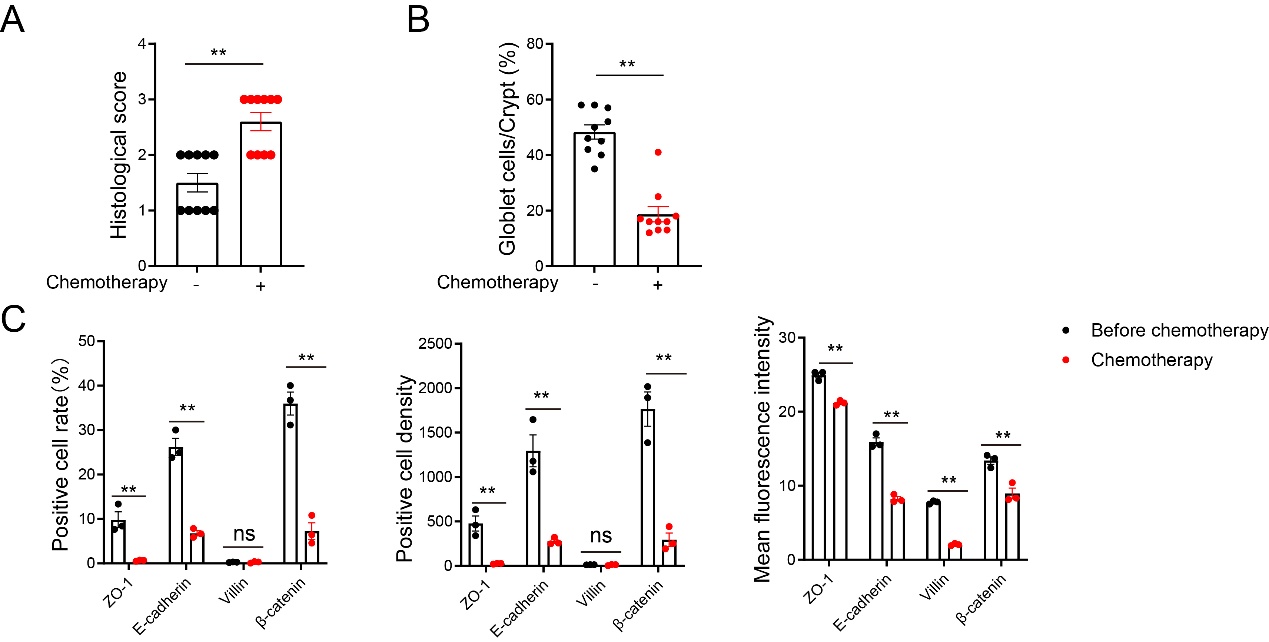
**

**Fig. S1. MIG ameliorates intestinal barrier dysfunction induced by 5-FU in a tumor-bearing mouse model.
(A)** Histological score of Fig. 1A.**(B)** PAS staining score of Fig. 1B. **(C)** Immunofluorescence score of Fig. 1C. Data are mean ± SEM; significance was determined by two-tailed unpaired *t*-test. **P* < 0.05, ***P*< 0.01 vs. normal group.

**
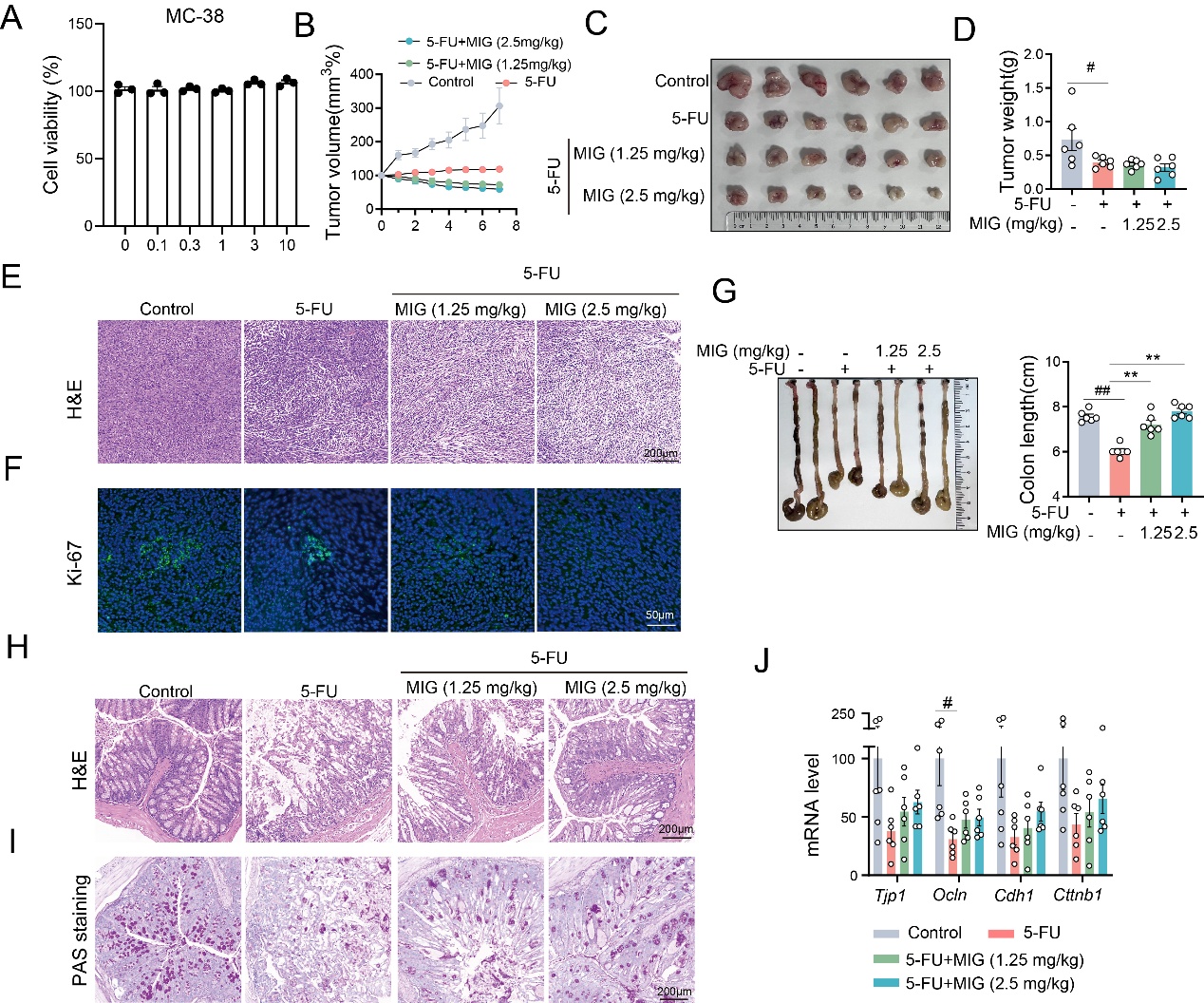
**

**Fig. S2. MIG ameliorates intestinal barrier dysfunction induced by 5-FU in a tumor-bearing mouse model.
(A)** Viability of MC-38 cells treated with MIG for 24 h.**(B-****J)** MC-38 tumor-bearing mice (n=6 per group) were treated with 5-FU (50 mg/kg, i.p., days 1–6) either alone or coadministered with MIG (1.25 or 2.5 mg/kg, i.p.). **(B)** Tumor volume measurements. **(C)** Representative tumor photographs. **(D)** Excised tumor weights. **(E)** H&E staining of tumor sections (scale bar = 200 μm). **(F)** Ki-67 staining of tumor tissues (scale bar = 50 μm). **(G)** Colon photographs and length measurements. **(H)** H&E staining of colon sections (scale bar = 200 μm). **(I)** PAS staining of colon tissues (scale bar = 200 μm). **(J)** qRT‒PCR analysis of *Tjp1*, *Ocln*, *Cdh1*, and *Ctnnb1* expression in colon of mice.

Data are mean ± SEM; significance was determined by two-tailed unpaired *t*-test or one-way ANOVA. ^#^*P* < 0.05 vs. normal group; **P* < 0.05, ***P*< 0.01, vs. Model group.

**
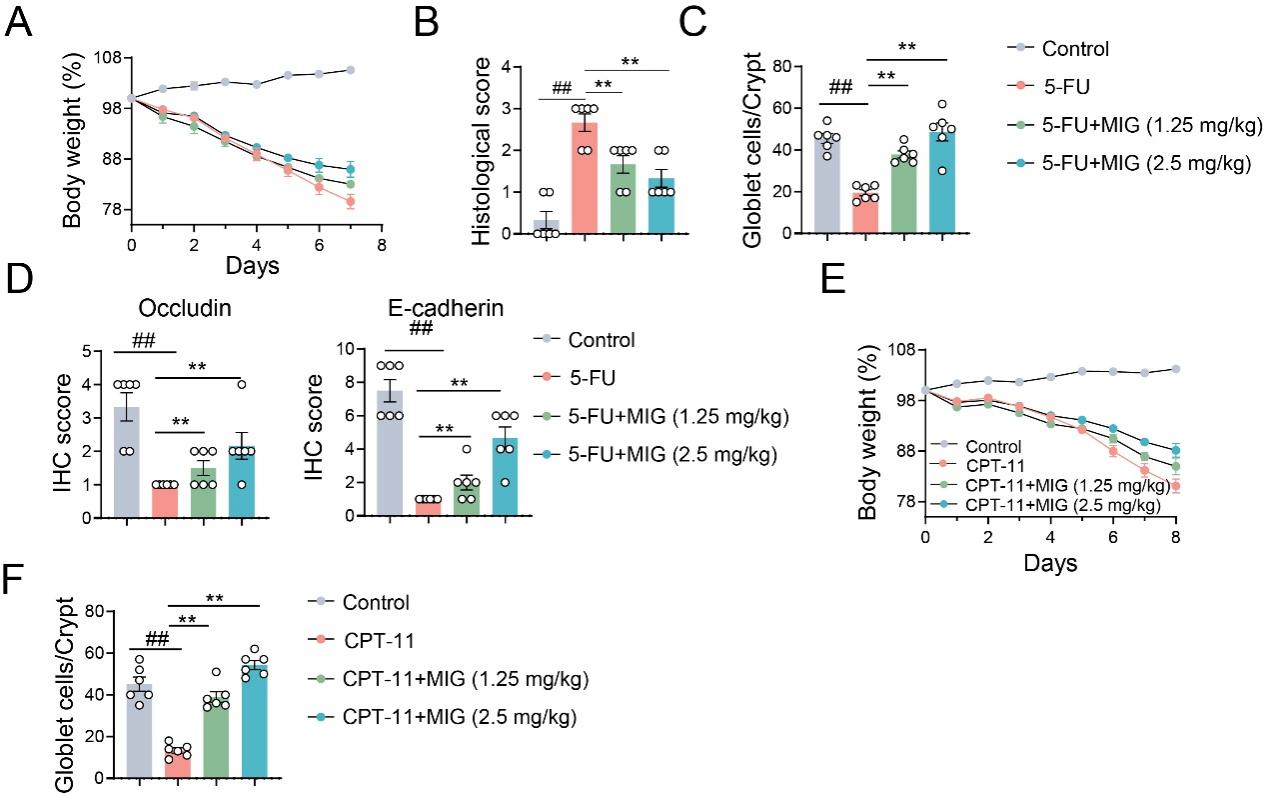
**

**Fig. S3. MIG ameliorated the intestinal injury induced by 5-FU and CPT-11. (A)** Body weight. (**B**) Histological scores of Fig.2C.**(C)** PAS staining score of Fig.2D. **(D)** Immunohistochemical staining score of Fig.2E. (**E**) Body weight. (**F**) PAS staining score of Fig.2J. Data are mean ± SEM; significance was determined by two-tailed unpaired *t*-test or one-way ANOVA. ^#^*P* < 0.05 vs. normal group; **P* < 0.05, ***P*< 0.01, vs. Model group.


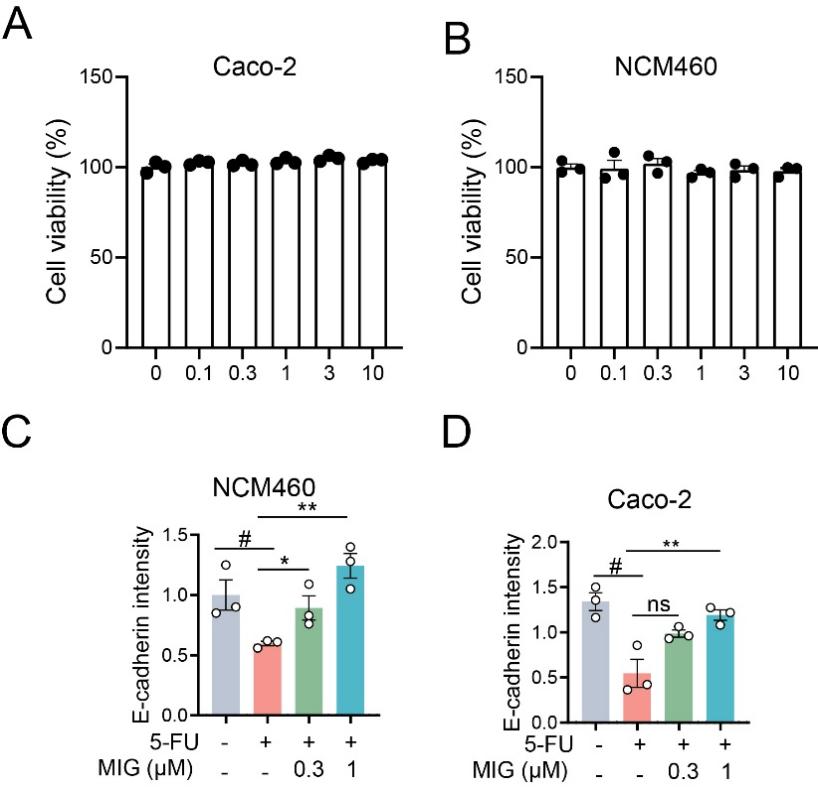


**Fig. S4. MIG increased E-cadherin expression upon treatment with 5-FU.** The viability of Caco-2 (**A**), NCM-460 (**B**) cells treated with different concentrations of MIG for 24 h. E-Cadherin expression in NCM-460 **(C)** and Caco-2 **(D)** cells shown in Fig. 3C. The data are presented as the means ± SEMs and are representative of three independent experiments. Statistical significance was assessed by a two-tailed unpaired *t* test. ^#^*P* < 0.05 vs. normal group; **P* < 0.05, ***P*< 0.01, vs. 5-FU group.


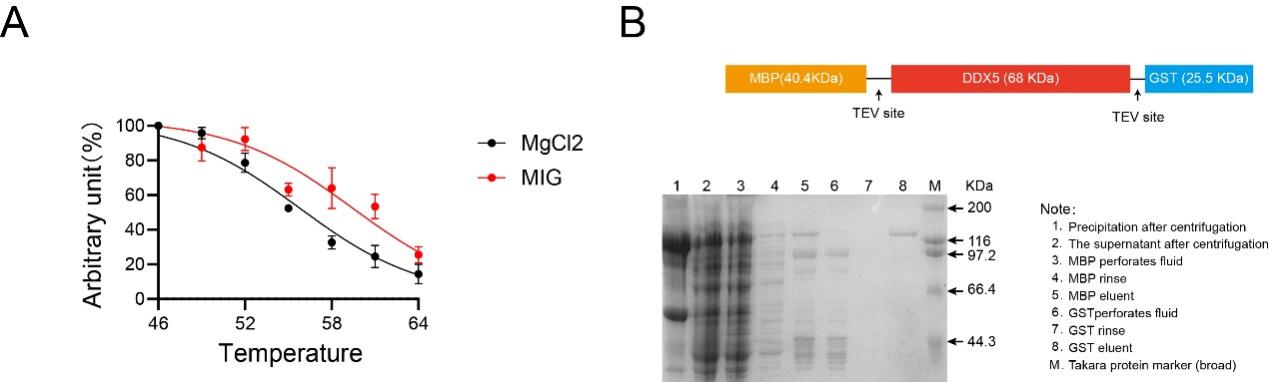


**Fig. S5. MIG binds to DDX5 directly**. (A) CETSA analyzed the thermal stabilization of PKM2 protein at different temperatures. (B) The expression of recombinant DDX5 protein. The recombinant DDX5 construct retains both affinity tags, with N-terminal maltose-binding protein (MBP, 40.4 kDa) and C-terminal glutathione S-transferase (GST, 25.5 kDa) fusion domains. The total molecular weight of this un-cleaved fusion protein is 133.9 kDa.


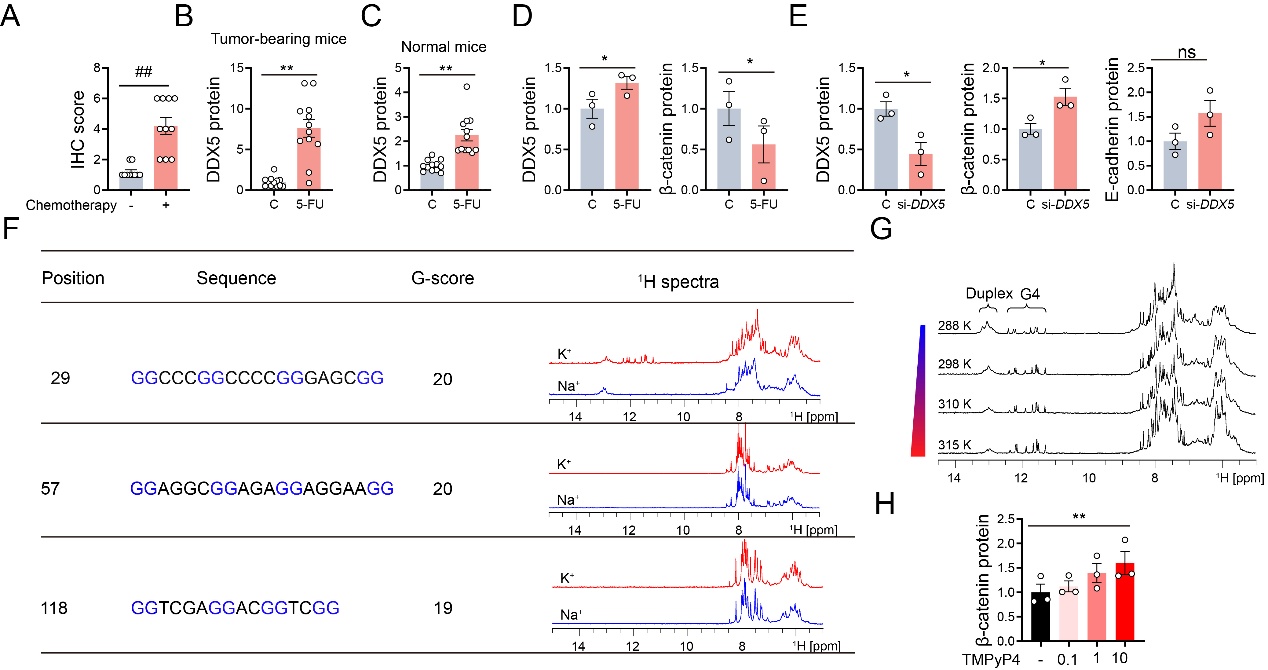


**Fig. S6. DDX5 unwind the G4 of *CTNNB1*.** **(A)** IHC analysis of DDX5. **(B)** Quantitative analysis of DDX5 in Figure 6B. **(C)** Quantitative analysis of DDX5 in Figure 6C. **(D)** Quantitative analysis of DDX5 (L) or β-catenin (R) described in Figure 6D. **(E)**Quantitative analysis of DDX5 (L) or β-catenin (M) or E-cadherin (R) described in Figure 6E. **(F)**Screening the G4 structure of *CTNNB1*. First, QGRS Mapper software was used to predict the DNA sequences in the 5'-UTR of the CTNNB1 gene that might form G4. Three sequences that could form G4s at the indicated locations (the Gs involved in the G4 core are marked in blue). Then, one-dimensional 1H NMR spectra were collected under Na+ and K+ conditions using WaterLOGSY NMR. The results showed that only position 29 could fold into a G4 structure under K+ conditions. The NMR acquisition conditions were as follows: 298 K, 0.2 mM DNA, 20 mM Tris-HCl (pH 7.5), 100 mM NaCl/KCl, and 10% D2O. **(G)** Variable temperature experiment. To observe the stability of the G4 structure formed by the position 29 sequence, the 1H NMR spectra were measured at different temperatures. The results showed that the G4 structure could stably form at temperatures ranging from 288 K to 315 K (increasing temperature facilitated the transformation from a double-stranded structure to the G4 structure), indicating that this G4 structure could stably exist at human body temperature. The NMR acquisition conditions were as follows: 288 K–315 K, 0.2 mM DNA, 20 mM Tris-HCl (pH 7.5), 100 mM KCl, and 10% D2O. **(H)** Quantitative analysis of β-catenin expression, as described in Fig. 2M. The data are presented as the means ± SEMs and are representative of three independent experiments. Statistical significance was assessed by a two-tailed unpaired *t* test. ^#^*P* < 0.05 vs. normal group; **P* < 0.05, ***P*< 0.01, vs. 5-FU group.


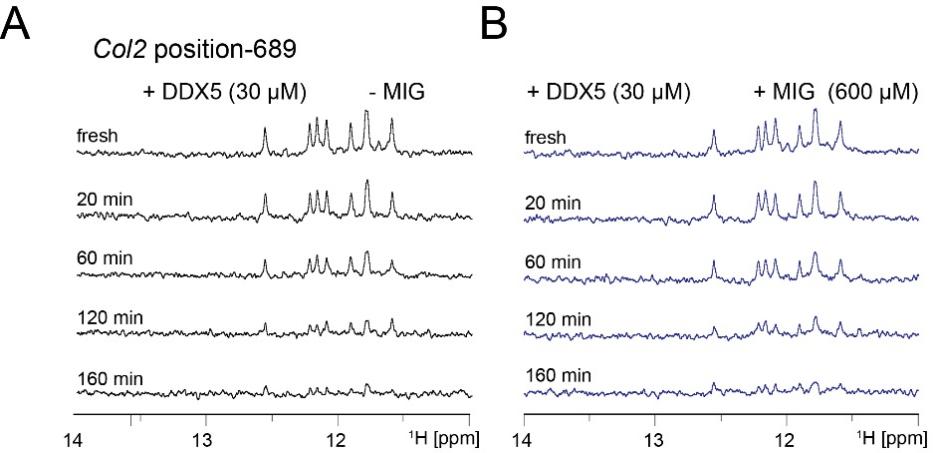


**Fig. S7. MIG did not affect the DDX5 unwinding of the *Col2* gene.** The position 689 region of the *Col2* gene promoter sequence was prefolded into a G4 structure, and then 30 µM DDX5 was added. **(A)** DDX5 could unwind this G4 structure. **(B)** The speed at which DDX5 unwound this G4 structure did not significantly change upon the simultaneous addition of 600 µM MIG to the system.


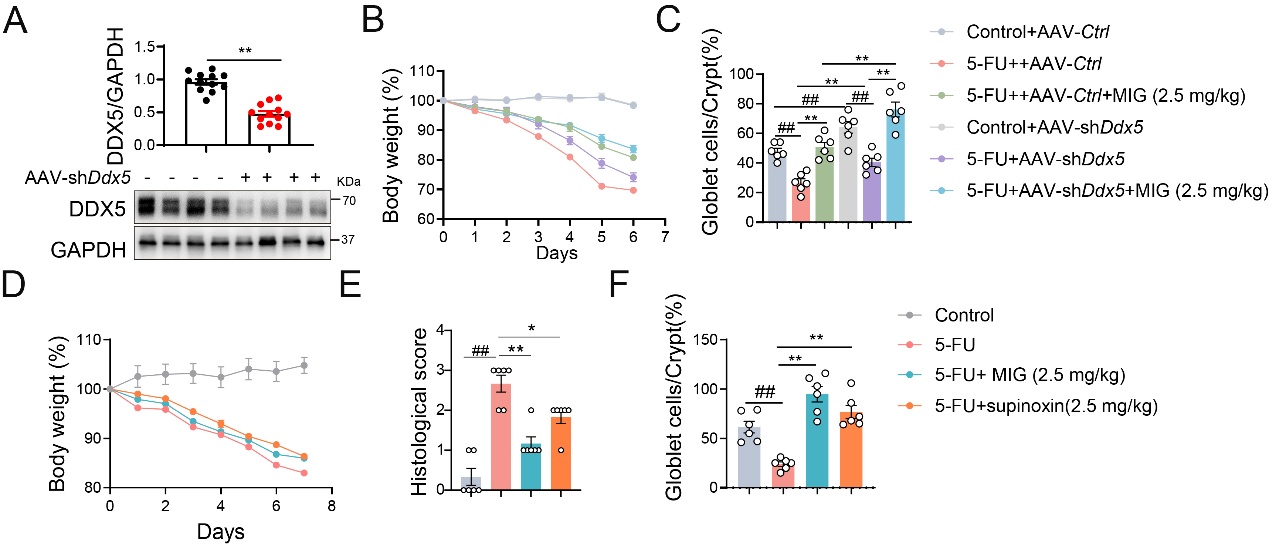


**Fig. S8. MIG maintain the intestinal epithelial barrier induced by 5-FU in *vivo*.**  (**A**) Western blot analysis of DDX5 expression levels in tissues of AAV-sh*Ddx5* mouse models. (**B**) Body weight of AAV-sh*Ddx5* *model*. (**C**) PAS staining score of Fig.7E. **(D)** Body weight of *Supinoxin model.* (**E**) Histological score of Fig.7I. (**F**) PAS staining score of Fig.7J. The data are presented as the means ± SEMs, statistical significance was assessed by a two-tailed unpaired *t* test. ^#^*P* < 0.05 vs. normal group; **P* < 0.05, ***P*< 0.01, vs. 5-FU group.
